# Hospital Expenditure.—The Commissariat

**Published:** 1896-07-11

**Authors:** 


### THE INSTITUTIONAL WORKSHOP.

### HOSPITAL EXPENDITURE.—THE COMMISSARIAT.

### XIX.—IS FOREIGN MEAT AN ECONOMY?

Although variations in the price of meat cannot be shown to account for the wide differences in the cost of this article in hospitals compared one with another, there can be no doubt that any such variation occurring in any one institution, say between one year and another, would be instantly reflected in the butcher's bill. Let a large institution change their existing meat contract for one quoting a price 2d. above or below what is now paid, and supposing that both contractors have been honest men, the close of the year will inevitably reveal a difference amounting to hundreds of pounds. Hence it is of great importance to arrive at some measure of truth respecting the use of foreign meat, which is certainly at least 3d. per lb. cheaper than English meat, and is now used in many hospitals, both for patients and staff.

Among the *idées fixes* most prevalent on this subject are the following:—

1. That foreign meat is more wasteful than English, that it diminishes in weight more in proportion during the cooking process, has more refuse parts which cannot be consumed, and less dripping and gravy.

2. That foreign meat contains a smaller proportion of nutritive qualities, is inferior altogether in food value, and is harder of digestion.

3. That foreign meat yields very unsatisfactory results when used in the preparation of beef tea; that the liquid is lighter in colour than that made from English meat, and contains a larger proportion of gelatine and less albumen.

It seemed likely that some of these assertions might easily be tested, and, accordingly, experiments have been made with a view to ascertaining as far as possible the position in which the foreign meat consumer stands with regard to the economy of his kitchen, of his digestive organs, and of his vital powers.

Isolated experiments can, of course, only partially elucidate truth in any matter. It is always arguable that though certain results have been obtained from a given selection of meat, quite contrary results might follow further experiments in the same line. Where, however, as in the present case, extreme care has been taken in the selection and testing of the joints, there is a strong presumption in favour of the general tendency revealed by the result. More than this cannot be claimed.

With regard to the first point—the general wastefulness of foreign as compared with home-killed meat—the following test was made. Two legs of mutton, under the direction of a clever practical cook, of nearly equal weight, were baked for an equal length of time in ovens equally heated. No. 1 was English grown and killed, and cost 10d. per lb.; No. 2 was the best Canterbury frozen mutton, thawed at the butcher's, and sent in ready for use. When sufficiently cooked, both joints were removed from the oven, and the meat was carved in slices as for hospital diets; the meat thus available for actual consumption was then weighed, as was also the remainder, including bone and all waste. The bone was subsequently divested of all scraps by boiling and scraping, and was then again weighed. No water was added to the baking pans, and thus it was possible to measure exactly the juice which ran out from the meat. The gravy which ran into the dish during the carving was first measured, and, secondly, the gravy found under the congealed dripping. The dripping was then weighed by itself. The results were as follows:—

Time of baking, two hours and a quarter.

| | English. | New Zealand. |
|---|---|---|
| | lbs. oz. | lbs. oz. |
| Weight when delivered ... ... ... | 8 6 ... | 7 14 |
| Weight when taken from oven ... ... | 5 15 ... | 5 13 |
| Weight of slices suitable for hospital diets | 3 4 ... | 3 0 |
| Weight of bone and waste after carving ... | 2 5 ... | 2 5 |
| Weight of pure bone ... ... ... | 0 11 ... | 0 11½ |
| Weight of dripping (cold) ... ... ... | 0 12 ... | 0 15 |
| | Tablespoonfuls. | |
| Gravy in dish after carving... ... ... | 8½ ... | 5½ |
| Gravy under dripping ... ... ... | 2¼ ... | 4 |
| Total quantity of gravy ... ... ... | 10¾ ... | 9½ |

Contrary to expectation, it was found that the English meat lost more in proportion during baking, viz., 2 lbs. 7 oz., against 2 lbs. 1 oz. This can be accounted for by the supposition that the freezing process has a drying effect on the meat, and this inference it will be seen is borne out by subsequent experiments.

It was the more remarked, as during the time of cooking the foreign joint dropped 3 oz. more dripping, and also nearly two tablespoonfuls more gravy than the English. The increase in the amount of dripping must be attributed to the fact that the foreign joint was the fatter of the two. The English joint yielded 4 oz. more useful meat, which is about in proportion to the additional half-pound weight at the beginning. The weight of bone and waste remaining over after carving was identical, and the weight of pure bone almost so. The English meat yielded rather more gravy taken altogether.

It will hardly be disputed that had both joints been English or both foreign the results could hardly have worked out more evenly. It must again be repeated that one experiment cannot be regarded as conclusive, and should any reader be tempted to try conclusions on the same lines the writer will gladly welcome any additional evidence on the question. It is clear, however, even from one experiment, that the assertion that foreign meat is essentially and invariably more wasteful than English cannot be supported.

This cannot, then, be taken as the explanation why many hospitals paying from 3½d. to 5½d. per lb. for their meat supplies spend more on their butcher than other hospitals paying from 6d. to 8d. Not only does common-sense rebel against the conclusion that highly-priced meat is more economical, but the contrary is shown in the experience of several metropolitan and provincial institutions, who, combining low prices with clever household management, have succeeded in hitting the minimum of expenditure and in maintaining at the same time a high standard of comfort for all the inmates.

We have already pointed out that the attempt to lower prices below the fair market average can only succeed in defeating the aims of true economy by opening the door to dishonest practices; a contractor who deliberately undersells his competitors is not an honest man, and can recoup himself for the deficiency in price by many little ways hard for the uninitiated to fathom, but costing the hospital eventually far more than if a reasonable quotation had been accepted. More than one manager who has been puzzled to find the butcher's bill excessive, notwithstanding an extremely low contract, has found an immediate saving subsequent to a change of contractor and a rise in price.

But these remarks apply equally to the consumer of English and foreign meat. The tables of comparative expenditure in London and elsewhere on this item have shown conclusively that a hospital may show an economical butcher's bill on either kind of meat, and it seems clear that in dieting the inmates a given weight of the one will go as far as a given weight of the other. Domestic management lies, after all, at the root of the whole meat question. Difference of price, however large a factor it may be in accounting for discrepancies in the average cost, is yet inconsiderable compared with the difference in the amount consumed, which may result from the state of the meat as sent from the butcher, and the care which is taken in the subsequent cooking and distribution. Failure in these points, and not the use of foreign meat, will be found to account for the anomaly occasionally presented in hospital accounts of lowest prices co-existing with highest bills.

